# Differential Gene Expression in Bladder Tumors from Workers Occupationally Exposed to Arylamines

**DOI:** 10.1155/2021/2624433

**Published:** 2021-11-08

**Authors:** Ramya T. Kolli, Zongli Xu, Vijayalakshmi Panduri, Jack A. Taylor

**Affiliations:** ^1^Epigenetics & Stem Cell Biology Laboratory, National Institute of Environmental Health Sciences, NIH, Research Triangle Park, NC, USA; ^2^Epidemiology Branch, National Institute of Environmental Health Sciences, NIH, Research Triangle Park, NC, USA

## Abstract

Occupational exposure to the arylamines benzidine and *β*-naphthylamine increase bladder cancer risk up to 100-fold, making them some of the most powerful human carcinogens. We hypothesize that tumors arising in people with occupational exposures have different patterns of gene expression than histologically similar tumors from people without such exposures. In a case-case study, we compare gene expression in 22 formalin-fixed paraffin-embedded (FFPE) bladder tumors from men with high-level occupational exposure to arylamines to that in 26 FFPE bladder tumors from men without such exposure. Gene expression analysis was performed on the NanoString nCounter system using a PanCancer Progression Panel comprised of 740 cancer progression-related genes and a custom panel of 69 arylamine- and bladder cancer-related genes which were chosen from *in vitro* studies. Although fold differences were small, there was evidence of differential expression by exposure status for 17 genes from the Progression Panel and 4 genes from the custom panel. In total, 10 genes showed dose-response association at a *p* < 0.01, of which 4 genes (*CD46*, *NR4A1*, *BAX*, and *YWHAZ*) passed a false discovery rate (FDR) *q* value cutoff of 0.05 but were not significant after Bonferroni correction. Overall, we find limited evidence for differentially expressed genes in pathways related to DNA damage signaling and epithelial-to-mesenchymal transition (EMT).

## 1. Introduction

Bladder cancer is the sixth most common type of cancer in the United States with an estimated 81,400 new cases in 2020 [1, 2]. Environmental exposures, occupational exposures, and tobacco smoking are all known risk factors of bladder cancer [3]. Occupational exposure to arylamines used in the production of azo dyes for paper, textile, and leather industries is one of the most powerful examples of human chemical carcinogenesis [4–9], raising bladder cancer risks up to 100-fold [10, 11]. Production of two key arylamines, benzidine and *β*-naphthylamine, has been banned in the United States since the early 1970s. In addition to occupational exposures, arylamines are one of the many carcinogenic compounds found in tobacco smoke [12, 13], with cigarette smoking increasing bladder cancer risk 2- to 3-fold [14, 15].

Benzidine forms DNA adducts and is mutagenic [16], leading to the generation of reactive oxygen species [17, 18], and it can induce chromosomal aberrations [16], leading to the induction of genes involved in DNA repair, apoptosis, and cell cycle arrest [19]. Exposure of human uroepithelial and bladder cancer cell lines leads to induction of genes involved in epithelial-to-mesenchymal transition (EMT) [20, 21]. We have previously investigated *p53* mutational patterns in bladder tumors from men with occupational exposure to arylamines compared to tumors from men without such exposure [22]. Here, we extend work with the same tumors to investigate expression differences in genes related to DNA damage, repair, cancer progression, and EMT.

## 2. Materials and Methods

### 2.1. Study Samples

Formalin-fixed paraffin-embedded (FFPE) blocks of transitional cell carcinoma of the bladder from men who had undergone surgical resection were retrieved from both occupationally exposed and unexposed groups as described in detail previously [22] and stored at room temperature. Normal bladder tissue was not available for study. The study was originally approved under the National Institutes of Health Clinical Project number 87-E-34, but all study subjects are now deceased and additional clinical information is not available.

Microtome sections (10 *μ*m thick) of the blocks were created for RNA extraction with corresponding serial sections. The sections were assessed for tumor characteristics including histological type, grade (using WHO classification), and extent of invasion. Patient and tumor characteristics are depicted in Supplementary Table S1.

The arylamine exposure score for exposed cases was calculated by multiplying the number of months a person worked in a given job/location with its corresponding level of exposure and summing it over the entire job history as previously described [9]. We categorized the unexposed subjects as group 0 (exposure score = 0, *n* = 26) and exposed workers into three groups based on tertiles defined in the original study [9]: the low-exposure group 1 (exposure score under 332; *n* = 9), the moderately high-exposure group 2 (exposure scores from 333 to 554; *n* = 8), and the heaviest-exposure group 3 (exposure scores over 554; *n* = 5).

### 2.2. RNA Extraction and Purification

Tumor sections were placed into Eppendorf tubes and deparaffinized using 1 ml of the HistoClear reagent (National Diagnostics, Atlanta GA) and heated for 12 minutes at 50°C. Samples were centrifuged at 800 rpm for 5 minutes at room temperature, and the supernatant was removed. The remaining pellet was washed twice with 1 ml of 100% ethanol, vortexed and centrifuged at room temperature for 2 minutes at maximum speed, and the ethanol was removed carefully without disturbing the pellet. The pellet was dried at 30°C for 30 minutes. Sequential isolation of RNA from FFPE tissue sections was performed using the MagMAX™ FFPE DNA/RNA Ultra Kit (Thermo Fisher Scientific, Waltham MA) following the manufacturer's instructions. RNA was quantified using the Qubit Fluorometer (Thermo Fisher Scientific, Waltham MA). RNA fragment sizes were analyzed using Experion™ Automated Electrophoresis System (Bio-Rad Laboratories Inc., Hercules CA).

### 2.3. Gene Expression Panels

The nCounter® PanCancer Progression panel (NanoString Technologies Inc., Seattle WA) is comprised of 740 genes related to cancer progression processes including angiogenesis, extracellular matrix remodeling (ECM), EMT, tumor growth, and metastasis. Details for the probes included in the Progression panel are available on request from NanoString Technologies Inc. using the form at http://www.nanostring.com/support-documents/ncounter-pancancer-progression-panel-gene-list/. We also designed a custom panel of 69 genes related to arylamine exposure and bladder cancer plus 30 housekeeping genes, 8 negative controls, and 6 spike-in hybridization/positive controls overlapping with those in the Progression panel. The custom genes and their annotations are listed in Supplementary Table S2. Genes in the custom panel were selected from *in vitro* and *in vivo* arylamine exposure studies [19–21, 23, 24] and include 25 genes related to the DNA damage signaling pathway [19, 23] and 4 genes related to EMT [20, 25]. We also included several genes related to bladder cancer from The Cancer Genome Atlas (TCGA) [26–28], which are highlighted in the Supplementary Table S2.

### 2.4. Gene Expression Analysis

An RNA template containing an estimated 200 ng of ≥ 300 nt fragments was used for gene expression analysis in nCounter NanoString assays. RNA sample preparation, hybridization, detection, and scanning with the nCounter SPRINT profiler, were performed following the manufacturer's instructions (NanoString Technologies Inc., Seattle WA). Results in the form of reporter code count (RCC) files were analyzed using nSolver™ Analysis Software following instructions for the Advanced Analysis add-on feature (NanoString Technologies Inc., Seattle WA). The raw NanoString counts were normalized using positive-control probe sets across all samples followed by biological normalization using the 30 housekeeping genes. Normalized data were log_2_ transformed and then used as input for differential expression analysis.

### 2.5. Differential Expression Analysis

Genes were excluded from differential analysis if expression levels in at least (≥) 20% of samples were less than the average values of the negative internal array controls (275 genes from the Progression panel and 26 genes from the custom panel). Gene expression differences in arylamine-exposed and unexposed bladder tumors were examined using robust linear regression models with *p* < 0.01 being considered as indicative of a statistically significant difference. Dose-response relationship between gene expression and arylamine exposure level was assessed using a robust linear trend test model with exposure level as a categorical variable and an FDR *q* value cutoff of 0.05. All tests were performed using R version 3.6.

## 3. Results

### 3.1. Patient Characteristics

Details for case samples are provided in Supplementary Table S1. The average age at surgery for arylamine-exposed cases (*n* = 22) was 67.5 years and that for unexposed cases (*n* = 26) was 68.8 years. All the subjects were white males.

### 3.2. NanoString Data Quality Control

For the Progression panel assays, all samples passed the default NanoString QC parameters including a binding density of 0.1–1.8, an image quality of 75%, a positive-control linearity of at least 0.95, and a limit of detection of 0.5 fM [29]. For the custom panel assays, 12 samples from unexposed men failed QC and were excluded from analysis. Positive and negative internal array controls in both the Progression and the custom panels generated expected signals with positive controls showing dilution linearity and negative controls showing minimal background signal (Supplementary Figures S1A and B). Additionally, for samples that passed QC, the total signal intensities for the 30 housekeeping genes on the two panels were well correlated with an *r*^2^ value of 0.8854 (Supplementary Figure S1C).

### 3.3. Arylamine Exposure-Associated Differential Expression

Gene expression analysis using the Progression panel identified 17 genes with differential expression between arylamine-exposed and unexposed cases (*p* < 0.01). Analysis of data from the custom panel identified 4 differentially expressed genes with *p* < 0.01 (Table 1). Among the 21 differentially expressed genes, we observed an increased expression of 13 genes and a decreased expression of 8 genes in arylamine-exposed cases compared to unexposed cases. Of these, only 10 genes from the Progression Panel and none from the custom panel had fold changes greater than 1.5, and none of the differentially expressed genes were significant after Bonferroni correction for multiple testing.

**Figure 1:**
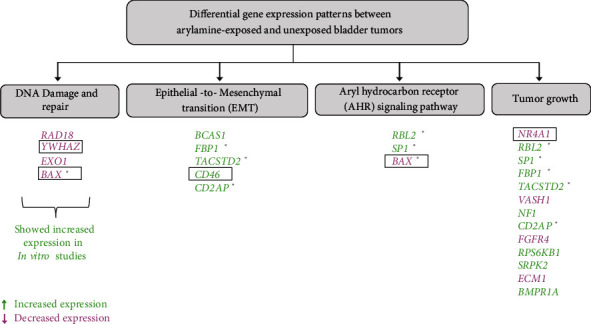
The summary of analysis results shows various relevant pathways highlighting differentially expressed genes related to these pathways. Genes highlighted in green depict increased expression and those highlighted in red depict decreased expression. ^∗^Genes related to more than one pathway. Genes that showed dose-response association with arylamine exposure are depicted in rectangular boxes.

A robust linear model was used to test the dose-response relationship between gene expression and arylamine exposure level. Within the 21 differentially expressed genes, 10 genes showed dose-response association at *p* < 0.01; of these, 4 genes (*CD46*, *NR4A1*, *BAX*, and *YWHAZ*) passed a false discovery rate (FDR) *q* value cutoff of 0.05 (Table 2). We observed that a positive correlation between arylamine exposure level and expression was present for *CD46*, whereas negative correlations were present for *NR4A1*, *BAX*, and *YWHAZ*. The summary of the results is shown in Figure 1.

Although normal bladder tissue was not available for this study, we made in silico comparison using the Michigan Portal for the Analysis of NGS Data (MiPanda) [30] and observed that most of the genes have similar expression levels in the bladder compared to other tissues, with 4 genes (*LRG1*, *VASH1*, *FGFR4*, and *EXO1*) expressed at somewhat lower levels and 4 genes (*BCAS1*, *NR4A1*, *FBP1*, and *TACSTD2*) at somewhat higher levels in the bladder than many other tissues. None of the genes are overexpressed in the bladder.

## 4. Discussion

Bladder cancer in workers occupationally exposed to arylamines has been investigated since the early 1900s, with substantial risks established in epidemiologic studies dating back to the 1950s [4, 7–9]. *In vitro* and *in vivo* exposure studies have evaluated gene expression changes associated with acute arylamine exposure [19, 20, 21, 23], whereas human occupation studies have often used blood DNA samples to examine genetic susceptibility and used tumor DNA samples to search for exposure-specific mutation signatures [22, 31–35]. Here, we extend that search by looking for exposure-specific gene expression signature.

Benzidine is a known genotoxic agent, and *in vitro* studies have shown that acute exposure to benzidine triggers a variety of checkpoint DNA damage and repair pathways and EMT in response to acute exposure [18–21]. We examined a panel of these genes and found four genes that showed differential expression including *RAD18*, *EXO1*, *BAX*, and *YWHAZ*. Three of the genes, *RAD18*, *EXO1*, and *BAX*, had decreased expression in arylamine-associated tumors instead of increased expression as reported in acute *in vitro* exposure studies [19, 23]. *RAD18* is a DNA damage-sensing gene, and *EXO1* is a mismatch repair gene, both of which are known to have increased expression from acute exposure to benzidine [19, 23]. *BAX* is an apoptosis regulator [24, 36] in the aryl hydrocarbon receptor (AHR) pathway [25] that is known to have increased expression in zebrafish embryos acutely exposed to benzidine [23], and *YWHAZ* is related to G2/M DNA damage checkpoint regulation [37] without a prior association with benzidine exposure. In our study, both *YWHAZ* and *BAX* showed decreasing dose-response expression with increasing history of arylamine exposure.

We observed differential expression (at *p* < 0.01) of a number of genes known to induce EMT including *BCAS1*, *FBP1*, *TACSTD2*, *CD46*, and *CD2AP*. Among these, *CD46* showed evidence of dose-response with a positive correlation with arylamine exposure level. EMT is an essential process in embryogenesis, organ development, and wound healing [38]. During EMT, the epithelial cells lose their epithelial characteristics and acquire mesenchymal characteristics by losing cellular adhesion, increasing motility, and defiance of apoptosis via extensive transcriptional changes to numerous genes [38, 39]. These processes are key events in cancer initiation and progression, and EMT is critically involved in initiation of tumorigenesis, metastasis, and progression of bladder cancer [40]. Exposure to carcinogens is known to induce EMT, and *in vitro* studies suggest that acute exposure to benzidine can induce EMT [20, 21]. Although we find some evidence that genes involved in the EMT pathway have altered expression in arylamine-associated tumors, they are not the specific genes implicated from prior *in vitro* studies. It is not surprising that the genes involved in acute response to an exposure do not match the genes with expression differences in tumors from patients with and without occupational exposure. In addition to bladder cells, *in vitro* studies have utilized a variety of different cell types, the expression changes observed may be transient, and those transient changes may be unrelated to tumorigenesis. Even if acute changes in expression are related to tumorigenesis, those expression differences would not necessarily persist in clinically apparent tumors.

Study limitations include the small sample size and missing patient data on potential confounders like smoking and tumor characteristics. Overall, we found evidence that several genes were differentially expressed by exposure status in pathways related to DNA damage signaling and EMT. However, fold differences were small, and none of the differences were statistically significant after Bonferroni correction for multiple testing.

## Figures and Tables

**(a):** 

PanCancer Progression Panel					
Gene	Mean normalized expression ± SD		Fold change	*p*	*q*
	Unexposed (*n* = 26)	Exposed (*n* = 22)			
LRG1	2.25 ± 1.13	3.41 ± 0.83	2.11	1.55 × 10^−4^	0.048
BCAS1	3.30 ± 1.85	4.92 ± 1.76	3.24	6.37 × 10^−4^	0.082
NR4A1	5.53 ± 1.58	4.00 ± 1.97	0.30	1.20 × 10^−3^	0.082
RBL2	2.86 ± 0.73	3.44 ± 0.58	1.52	1.49 × 10^−3^	0.082
SP1	3.38 ± 1.18	4.24 ± 0.84	1.85	1.64 × 10^−3^	0.082
FBP1	2.99 ± 1.36	4.31 ± 1.50	2.31	1.84 × 10^−3^	0.082
EIF2AK3	2.56 ± 0.92	3.25 ± 0.91	1.78	1.86 × 10^−3^	0.082
TACSTD2	5.15 ± 1.95	6.74 ± 1.53	3.15	2.58 × 10^−3^	0.090
CD46	3.22 ± 0.88	3.89 ± 1.12	1.71	2.65 × 10^−3^	0.090
VASH1	3.78 ± 1.16	2.74 ± 1.17	0.52	2.91 × 10^−3^	0.090
NF1	3.05 ± 0.92	3.78 ± 0.76	1.65	3.67 × 10^−3^	0.096
CD2AP	2.59 ± 1.32	3.42 ± 0.96	1.97	3.69 × 10^−3^	0.096
FGFR4	3.28 ± 0.94	2.45 ± 1.24	0.57	3.99 × 10^−3^	0.096
RPS6KB1	3.29 ± 0.73	3.66 ± 1.01	1.50	5.76 × 10^−3^	0.120
SRPK2	4.05 ± 0.95	4.66 ± 0.66	1.46	5.79 × 10^−3^	0.120
ECM1	3.65 ± 1.02	2.80 ± 1.27	0.56	7.10 × 10^−3^	0.138
BMPR1A	5.10 ± 0.83	5.52 ± 0.95	1.48	8.34 × 10^−3^	0.153

**(b):** 

Custom panel					
Gene	Mean normalized expression		Fold change	*p*	*q*
	Unexposed (*n* = 15)	Exposed (*n* = 22)			
RAD18	5.49 ± 1.04	4.47 ± 0.68	0.53	1.44 × 10^−3^	0.040
YWHAZ	7.14 ± 0.88	6.23 ± 0.96	0.57	1.86 × 10^−3^	0.040
EXO1	2.85 ± 0.79	2.07 ± 1.51	0.55	3.04 × 10^−3^	0.044
BAX	5.49 ± 0.89	4.30 ± 1.44	0.47	5.38 × 10^−3^	0.058

**(a):** 

PanCancer Progression Panel							
Gene	Mean normalized expression ± SD				Coef	*p*	*q*
	Unexposed group (*n* = 26)	Exposure group 1 (*n* = 9)	Exposure group 2 (*n* = 8)	Exposure group 3 (*n* = 5)			
CD46	3.22 ± 0.88	3.52 ± 1.41	3.86 ± 0.71	4.79 ± 0.65	0.436	2.83 × 10^−5^	0.014
NR4A1	5.53 ± 1.58	4.33 ± 1.8	4.29 ± 2.24	2.67 ± 1.58	-0.899	1.39 × 10^−4^	0.017

**(b):** 

Custom panel							
Gene	Mean normalized expression ± SD				Coef	*p*	*q*
	Unexposed group (*n* = 15)	Exposure group 1 (*n* = 9)	Exposure group 2 (*n* = 8)	Exposure group 3 (*n* = 5)			
BAX	5.49 ± 0.89	4.52 ± 1.71	4.03 ± 1.47	4.28 ± 0.81	-0.485	1.34 × 10^−3^	0.048
YWHAZ	7.14 ± 0.88	6.46 ± 0.82	6.21 ± 1.12	5.73 ± 1.02	-0.377	2.33 × 10^−3^	0.048

## Data Availability

Additional data are available in the Supplementary material.

## Supplementary Materials

Supplementary Materials Figure S1: nanostring data quality control. Table S1: patient and tumor characteristics. Table S2: custom genes and annotations.
