# Supplementary material for: Differential Gene Expression in Bladder Tumors from Workers Occupationally Exposed to Arylamines
Source: Biomed Res Int. 2021 Nov 8;2021:2624433. doi: 10.1155/2021/2624433 (PMC8592720; doi:10.1155/2021/2624433)
Supplement: Supplementary Materials — Figure S1: nanostring data quality control. Table S1: patient and tumor characteristics. Table S2: custom genes and annotations. [file 2624433.f1.pdf]

SUPPLEMENTARY MATERIAL

Title: Differential Gene Expression in Bladder Tumors from Workers Occupationally Exposed to Arylamines

Authors: Ramya T. Kolli, Zongli Xu, Vijayalakshmi Panduri and Jack A. Taylor

Table S1: Patient and tumor characteristics.

Characteristic	Unexposed cases (n = 26)	Exposed cases (n = 22)
Mean age at surgery	68.8	67.7
Average exposure score	0	340.4
Tumor grade		
1	1	1
2	1	6
3	7	9
4	1	3
Missing data	16	3
Invasion		
Yes	9	14
No	4	8
Missing data	13	0
Tumor morphology		
Papillary	3	7
Nonpapillary	8	15
Missing data	15	0
Smoking status		
Current	3	8

Table S1 continued

Characteristic	Unexposed cases (n = 26)	Exposed cases (n = 22)
Former	7	4
Never	1	0
Missing data	15	10
Mean pack-years	44.74	23.81

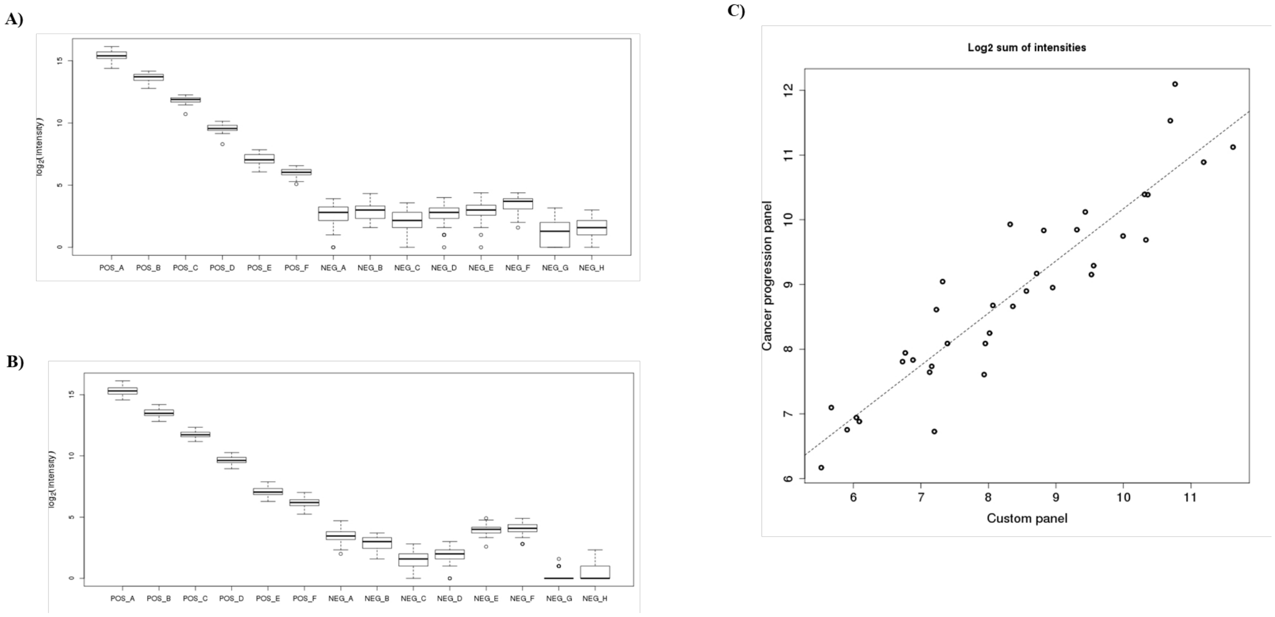

Figure S1. Boxplots of raw counts for positive controls and negative controls from **A)** nCounter Cancer Progression panel and **B)** Custom panel. Horizontal lines from bottom to top in each box indicate first quartile, median and third quartile. Whiskers indicate 1.5 times the interquartile range from the first and third quartile. **C)** Scatterplot shows the comparison of signal intensities for the 30 housekeeping genes between nCounter PanCancer Progression panel and the custom panel. The signals are represented as log_2 sum of intensities from the two panels ($r^2 = 0.8854$).
